# Effects of Antimony on Rice Growth and Its Existing Forms in Rice Under Arbuscular Mycorrhizal Fungi Environment

**DOI:** 10.3389/fmicb.2022.814323

**Published:** 2022-03-22

**Authors:** Min Zhou, Xinru Li, Xuesong Liu, Yidong Mi, Zhiyou Fu, Ruiqing Zhang, Hailei Su, Yuan Wei, Huifang Liu, Fanfan Wang

**Affiliations:** ^1^College of Environment, Hohai University, Nanjing, China; ^2^State Key Laboratory of Environmental Criteria and Risk Assessment, Chinese Research Academy of Environmental Sciences, Beijing, China; ^3^School of Ecology and Environment, Inner Mongolla University, Hohhot, China

**Keywords:** arbuscular mycorrhizal fungi, rice, antimony, uptake, transformation, biomass, antioxidant enzyme

## Abstract

Arbuscular mycorrhizal fungi (AMF) can form symbiotic relationships with most terrestrial plants and regulate the uptake and distribution of antimony (Sb) in rice. The effect of AMF on the uptake and transport of Sb in rice was observed using pot experiments in the greenhouse. The results showed that AMF inoculation increased the contact area between roots and metals by forming mycelium, and changed the pH and Eh of the root soil, leading to more Sb entering various parts of the rice, especially at an Sb concentration of 1,200 mg/kg. The increase in metal toxicity further led to a decrease in the rice chlorophyll content, which directly resulted in a 22.7% decrease in aboveground biomass, 21.7% in underground biomass, and 11.3% in grain biomass. In addition, the antioxidant enzyme results showed that inoculation of AMF decreased 22.3% in superoxide dismutase, 9.9% in catalase, and 20.7% in peroxidase compared to the non-inoculation groups, further verifying the negative synergistic effect of AMF inoculation on the uptake of Sb in rice. The present study demonstrated the effect of AMF on the uptake and transport of Sb in the soil–rice system, facilitating future research on the related mechanism in the soil–rice system under Sb stress.

## Highlights

- AMF should reduce the plant physiological character like decrease in chlorophyll and biomass under different concentration of Sb in the soil-rice system.
- AMF inoculation leading to more Sb entering various parts of the rice.
- Inoculation of AMF increased metal toxicity further led to a decrease in rice biomass.
- The activity of antioxidant enzyme decreased further verifying the negative synergistic effect of AMF inoculation on the uptake of Sb in rice.

## Introduction

Antimony (Sb) is a carcinogenic element. Excessive Sb exposure leads to serious health consequences for humans, causing damage to the respiratory, cardiovascular, and urinary systems (Schnorr et al., 1995). Therefore, Sb was listed as a priority pollutant by the European Union (Filella et al., 2002) and the Environmental Protection Agency (Wei et al., 2015). Moreover, the antimony compound was listed as group 2B by the International Agency for Research on Cancer (IARC) (Saerens et al., 2019), and a restrictive pollutant by China. At present, the main sources of Sb pollution are anthropogenic activities, such as mining, metallurgy, alloy, fireproof materials, and medicines (Fan et al., 2016). In China, Sb concentrations in the soil can reach 3,365–5,949.2 mg/kg in the surrounding area of Sb mines at LengShuiJiang, Hunan province (Li et al., 2018; Zhang Q.M. et al., 2020; Zhang Y.X. et al., 2020), and the Sb content in paddy soil surrounding Xikuangshan was over 1,500 mg/kg (Okkenhaug et al., 2012). Although Sb is a non-essential element to plants, Sb in the soil can readily accumulate in plants and enter the food chain. Rice is a staple food crop, providing for 3 billion people in the world (Ren et al., 2014). Therefore, rice safety is crucial to the global population. The World Health Organization (WHO) reported that rice is the major pathway for Sb to enter the food chain, accounting for 33% of the intake of Sb in the human body. Wu et al. (2011) reported that the Sb concentration of rice can reach up to 0.93 mg/kg near the XiKuangShan mine. The tolerable daily intake (TDI) of Sb near the XiKuangShan mine is 1.54-fold higher than the WHO recommended value (Wu et al., 2011). Therefore, research on rice uptake and transport of Sb in soil–rice systems for food safety has become particularly important.

Microorganisms are essential components in soil–plant systems. The interaction between microorganisms and plant is important component in ecosystem, and was considered as an important partner that regulate local and systemic mechanisms in plant (Meena et al., 2017). Therefore, it is inevitable to consider the effect of microorganisms on the uptake and transport of Sb in soil–rice systems. AMF are a category of beneficial microorganisms in which all species identified belong to *Glomeromycota* (Redecker et al., 2013), and AMF can form symbiotic relationships with more than 80% of terrestrial plants (Wang and Shi, 2008). A large number of papers have reported AMF can form symbiosis with rice (Chen et al., 2017; Parvin et al., 2019). For example, Parvin et al. (2019) used high throughput Illumina sequencing found that there were 77 operational taxonomic units (OTUs, based on a sequence similarity threshold of 97%) from eight AMF families from 45 rice fields. In addition, AMF can form symbioses with plants when the plants are exposed to excessive Sb (Wei et al., 2015; Pierart et al., 2018; Xi et al., 2021). AMF accelerate the growth of plants by improving essential mineral element uptake, changing the root structure of host plants, and increasing heavy metal resistance (Solís-Domínguez et al., 2011; Hernández-Ortega et al., 2012). For instance, He et al. (2014) observed that inoculation with *Glomeraceae* had a significantly positive effect on plant growth, especially at high concentrations of heavy metals, compared to plants not inoculated with *Glomeraceae*. Furthermore, there are numerous reports on the effect of AMF on the uptake of Sb in plants. For example, under Sb exposure, AMF improved Sb absorption in carrots (Pierart et al., 2018) and *Cynodon dactylon* (Wei et al., 2016), whereas the opposite result was observed in maize (Shen et al., 2017). However, only a few studies have reported the effect of resistant bacteria on the uptake of Sb from soil–rice systems. For instance, Long et al. (2020) reported that an Sb-resistant bacterium can alter the iron plaque distribution of rice roots thus affect the uptake of Sb by rice, and Sun et al. (2019) found that when rice was exposed to antimony, different flooding conditions resulted in different microbial community structures. Therefore, based on above analysis, we speculated that AMF may be formed symbiotic relationship with rice, and, affect rice on Sb uptake and distribution.

To investigate the effect of AMF on the uptake of Sb in rice, we designed a comparative experiment of AMF inoculation with non-inoculated tests and measured chlorophyll, antioxidant enzymes, and Sb adsorption in different parts of rice plants using inductively coupled plasma mass spectrometry (ICP-MS, Agilent 7500, Agilent Technology, United States), the chlorophyll meter (SPAD-502 Plus, Tuo Pu, China), and ultraviolet and visible spectrophotometer technology (Agilent 8453, Agilent Technology, United States). The aim of this study was to: (1) measure biomass and chlorophyll to elucidate the effect of AMF on growth and physiology; (2) determine the effect of AMF on the chemical properties of rhizosphere soil by measuring pH and redox potential (Eh); (3) determine the effect of AMF on the uptake of Sb by measuring the Sb concentration of different plant parts and Sb speciation; and (4) further analyze the activities of several typical antioxidant enzymes to evaluate the effects of AMF on the accumulation of Sb in rice. These results will further reveal the distribution and morphology of Sb in rice under the presence of AMF, which will help us better understand the migration and transformation of Sb in soil-rice system after AMF inoculation and the related effects on food crops.

## Materials and Methods

### Soil, Fungi, and Plants

Soil was collected from Hunan Agricultural University in Hunan Province, China (113°5′23″E, 28°11′24″N). It was air dried and passed through a 2-mm sieve. The soil was then sterilized by autoclave steam for 2 h at 121°C under 0.1 MPa pressure. Four soil Sb concentrations (0, 300, 600, and 1,200 mg Sb/kg) were prepared by adding an appropriate volume of potassium pyroantimonate [KSb(OH)_6_] stock solutions (0, 300, 600, and 1,200 mg Sb/kg as K_2_H_2_Sb_2_O_7_⋅4H_2_O in ultrapure water) to the soil and then adding ultrapure water (Millipore-Q water, 18 Ω⋅cm) to maintain field capacity. The soil was aged for 4 weeks before being used in the experiment.

AMF (*Glomus mosseae*, BGC NM01A) was obtained from the Beijing Academy of Agriculture and Forestry Sciences, which contained the spores and hyphae of AMF and the rhizosphere soil of cultivated AMF.

Rice seeds (*Oryza sativa* L., Xiangwanxian No. 12) were purchased from the Hunan Rice Research Institute. They were surface-sterilized by soaking in 10% H_2_O_2_ solution for 20 min and then rinsed with ultrapure water five times to clear the residual H_2_O_2_. The seeds were wrapped in aseptic wet gauze placed in the FPQ multi-stage artificial climate box for 3 days in the dark for germination. The wet gauze was changed every 6 h. The germinated seeds were transferred to a 5.4-L polyvinyl chloride plate with 32-orifices containing aseptic soil substrate in the FPQ multi-stage artificial climate box and were cultured for 3 weeks. For 1 week, 10 mL 0.5-strength Hoagland nutrient solution was added to the orifices at the three leaf stage; thereafter, full strength Hoagland nutrient solution was used (Ren et al., 2014). The ratio of the light-to-dark cycle was 14–10 h with 180–240 μmol/(m^2^⋅s) sodium light. The temperature of the light period and that of the dark period were kept at 27 ± 1 and 20 ± 1°C, respectively. The relative humidity was maintained at 65–70%.

### Pot Experiment Design

In the pot cultural experiment, rice was selected as the host plant, and *Glomus mosseae* was used as the inoculum. Two series of soil, which had four Sb concentrations (0, 300, 600, and 1,200 mg/kg) in each series, were prepared and used for rice growth. For each Sb concentration, three parallel pots, with each pot containing 5 kg of soil, were employed. The first series was inoculated with inactive AMF (M−); the second series was inoculated with active AMF (M+). The AMF in the first series was inactivated by autoclaved steam for 2 h at 121°C under 0.1 MPa pressure and then put into each pot. The rice seedlings at the four-leaf stage of a similar size and shape from culturing were selected and planted in each pot. Three seedlings were planted in each experimental pot. The base fertilizer was composted of CO(NH_2_)_2_, Ca(H_2_PO_4_)_2_, and KCl, and the respective rates were 1, 1, and 1.5 g/kg soil (He and Yang, 1999). The experiments were conducted in the greenhouse of the Chinese Research Academy of Environmental Sciences in Beijing. After 120 days, the rice plants were harvested.

### Infection of Rice by Arbuscular Mycorrhizal Fungi

AMF infection of rice root was determined after 30 days of transplanting. The method of infection was measured according to Vierheilig et al. (2005) with some modifications. The fresh roots were cleared with deionized water, and then cut into 2-cm root segments. Cleared roots were added into stationary liquid for 24 h which contained formaldehyde, acetic acid and 50% ethanol, and the volume of rates were 13, 5, and 200, respectively. After cleaned, the root segments were transferred to the 50-mL beaker which contained 10% KOH. Then the beaker was in water bath at 90°C for root segments transparent. 5% acetic acid were added to root segments for acidification. The root segments were stained with 5% ink-vinegar for 5 min and cleared with tap water which contained several drops acetic acid. The root segments were then transferred to glass slide and added 2 drops of lactic acetic acid. The root segments were observed under a microscope (LIOO JS-750T, Germany).

### Biomass of Rice and Chlorophyll Content of Rice Leaves

After harvesting, rice plants were rinsed with deionized water five times. The plants were cut into roots, stems, leaves, and grain by ceramic scissors and then dried in an oven for 72 h at 65°C. All parts of the rice plant were weighed with an electronic balance. Each part was weighed three times, and the average was taken.

The middle of the sixth top leaf was used to measure the chlorophyll content with a chlorophyll meter. Each part was measured three times, and the average was taken.

### Antimony Concentration in Rice and Antimony Speciation in Rice Plants

The roots, stems, leaves, and grain of rice were freeze dried with a FD5-series freeze dryer (SIM, United States), and 100 mg of each sample was transferred to a digestion vessel containing 2 mL HNO_3_. The digestion vessel was sealed and digested in a microwave instrument (CEM, United States) for 2 h according to the digestion procedure (Supplementary Table 1), after cooling to room temperature. The digestion tubes were opened and transferred to a water bath (90°C) until the digestion solution became clear. Then, it was cooled to an ambient temperature. The solution was diluted with 1% HNO_3_ to 50 mL and filtered through a 0.45-μm polyether sulfone membrane before being analyzed by ICP-MS. The certified reference material, tomato leaves (ESP-1, China National Environmental Monitoring Center reference material), was used for quality control.

The method for measuring Sb speciation in rice plants was done according to Okkenhaug et al. (2012) with some modifications. After being frozen and dried, the rice roots were cut into fragments with ceramic scissors. Plant samples (0.3000 g) were weighed into a 5-mL centrifuge tube containing 3 mL of 100 mM citric acid. The solutions were oscillated for 30 min at 50°C with a vortex centrifuge and centrifuged at 1,033 × g for 10 min. The supernatants were extracted once again with the above method. The extraction solutions of the two supernatants were filtered with a 0.45-μm polyether sulfone membrane and stabilized with 10 mL of citric acid. The solutions were measured by ICP-MS.

### Soil pH and Redox Potential

The soil samples were air-dried, ground to a power with a quartz mortar, and filtered through a 2-mm sieve. The soil pH was determined based on a soil-to-deionized water ratio of 1:2.5. The soil–water mixture was stirred for 5 min and settled for 30 min. The supernatant was then measured with a calibrated pH meter (PHBJ-260, Lei Ci, China). The results were replicated three times.

The Eh value for rhizosphere soil was measured following the method previously described by Chen et al. (1997) with a platinum electrode. Briefly, the platinum electrode (0.5-mm diameter) was inserted to a depth of 5 cm within the rhizosphere soil to measure the Eh value at different positions in each pot. Each pot was measured three times, and the average was calculated.

### Activity of Antioxidant Enzymes and Malondialdehyde Content

To prepare the enzyme solution, 3 g fresh rice leaves were added to a glass mortar containing 30 mL phosphate buffer (pH 7.8) at 4°C. The mixture was homogenized and transferred into a 50-mL centrifuge tube. This was followed by centrifuging for 15 min at 1,837 × g. The supernatant was collected and transferred to a 150-mL volumetric flask. The centrifugation process was then repeated once. Ultrapure water was used to obtain a solution volume of 150 mL in the volumetric flask. This solution was used to determine enzyme activities (superoxide dismutase, SOD; peroxidase, POD; catalase, CAT) and the content of malondialdehyde (MDA).

### Superoxide Dismutase Assay

The SOD assay was performed according to Giannopolitis and Ries (1977). Briefly, 3 mL of SOD test solution were prepared by mixing 1.5 mL 0.05 mol/L phosphate buffer, 0.3 mL 130 mmol/L methionine (Met) solution, 0.3 mL 0.75 mmol/L nitroblue tetrazolium (NBT) solution, 0.3 mL 0.1 mmol/L disodium edetate dihydrate (EDTA-Na_2_) solution, 0.3 mL 0.02 mmol/L riboflavin, 0.05 mL enzyme solution, and 0.25 mL of deionized water. Phosphate buffer was used instead of enzyme solution for the control group. The solutions were kept in the dark and irradiated under 4,000 xl fluorescent lamps for 20 min. The absorbance was determined at 560 nm with an ultraviolet and visible spectrophotometer.

### Catalase Assay

The CAT assay was performed using the method of Knörzer et al. (1996). In brief, 2.5 mL enzyme solution and 2.5 mL 0.1 mol/L H_2_O_2_ were mixed in 50-mL triangular flasks. The solution was then heated in a water bath for 10 min at 30°C. After heating, 2.5 mL 10% H_2_SO_4_ was immediately added to the triangular flask. KMnO_4_ (0.1 mol/L) was used to titrate the solution in the triangular flask after the solution became colorless, and the number of burettes was recorded. The enzyme solution of the control group was inactivated. CAT was measured at a wavelength of 240 nm by an ultraviolet and visible spectrophotometer.

### Peroxidase Assay

To measure the activity of POD, 2.9 mL 0.05 mol/L phosphate buffer, 1.0 mL 2% H_2_O_2_, 1.0 mL 0.05 mol/L guaiacol, and 0.1 mL enzyme solution were added to a 10-mL test tube and immediately heated in a water bath for 15 min at 37°C. Then the test tube was immediately transferred to an ice bath, and 2.0 mL of 20% trichloroacetic acid (TCA) was added to terminate the reaction. This was followed by centrifuging at 2,871 × g for 10 min. The supernatant was collected and diluted with phosphate buffer to 20 mL. The activity of POD was measured at an absorbance of 470 nm by an ultraviolet and visible spectrophotometer. A control group was also prepared in the same procedure, except the enzyme solution was inactivated.

### Malondialdehyde Content

The MDA content was assayed using Chakraborty et al. (2013). Enzyme solution (0.5 mL) was added to the centrifuge tube containing 1 mL 20% trichloroacetic acid (TCA) and 0.5% thiobarbituric acid (TBA). The mixture was incubated for 30 min at 95°C and then stopped by placing the tubes in an ice bath. The mixture was centrifuged for 10 min at 11,487 × g (GTR21-1, China). The absorbance of the supernatant, measured by an ultraviolet and visible spectrophotometer at 600 nm, was subtracted from the absorbance at 532 nm.

### Statistical Analyses

The biomass, chlorophyll content, MDA content, Sb concentration in different parts, antioxidant enzymes, pH, and Eh data in rhizosphere soil were shown as the mean ± standard deviations (*n* = 3), except for the speciation of Sb in rice roots. All data were examined with one-way analysis of variance (ANOVA) combined with Student’s *t*-test (*P* < 0.05). The experimental data were analyzed with SPSS^®^ 21.0 (SPSS, United States) software. The graphs were plotted with Origin 9.1 (OriginLab, United States).

## Results and Discussion

### Effects of Arbuscular Mycorrhizal Fungi Inoculation on Biomass and Chlorophyll Content of Rice

**FIGURE 1:**
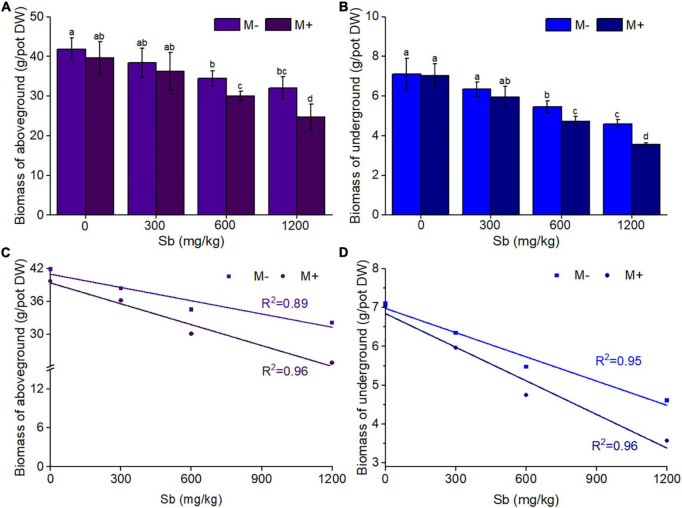
Effects of AMF on rice biomass of aboveground and underground. **(A)** Biomass of aboveground, **(B)** biomass of underground, **(C)** the liner relationship between biomass of aboveground and concentrations of Sb, **(D)** the liner relationship between biomass of underground and concentrations of Sb. DW represents dry weight. Error bar was calculated from three parallel samples. Error bars sharing no common letter indicate that biomass are significantly different at *P* < 0.05 level for treatments. The data are means ± standard deviations (SDs) (*n* = 3).

**FIGURE 2:**
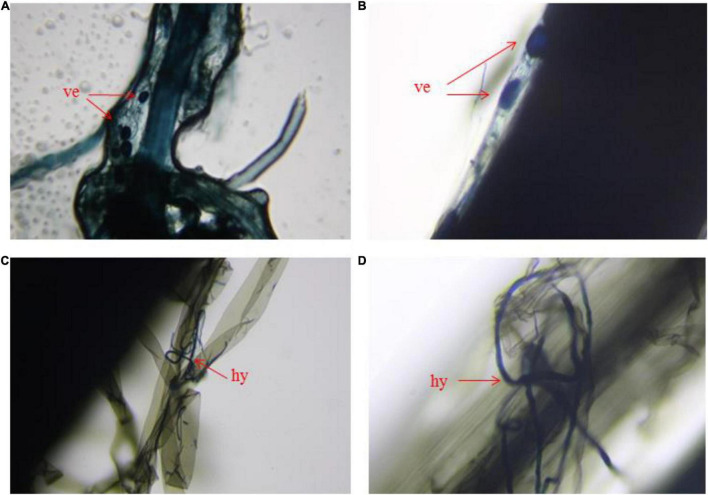
Microstructure of arbuscular mycorrhizal fungi infect the root of rice. **(A,B)** Vesicle in roots of arbuscular mycorrhizal fungi, **(C,D)** hypha of arbuscular mycorrhizal fungi in roots. ve represents vesicle, hy represents hypha.

In the same concentration of Sb polluted environment, the reduction of aboveground and underground biomass of rice in the AMF inoculated group was significantly enhanced compared with that in the AMF non-inoculated group (Figures 1A,B and Supplementary Table 2). As shown in Figure 1, AMF had little effect on both aboveground rice biomass and underground rice biomass without Sb contamination (concentration of Sb was 0 mg/kg). With the increase in Sb concentration, the negative effect of AMF on rice biomass gradually appeared and became more and more obvious, the same phenomenon can also be seen intuitively in Supplementary Figure 1. The degree of biomass reduction resulting from AMF inoculation was similar in both the aboveground and underground parts of rice. For example, AMF inoculated rice treated with a series dose (300, 600, and 1,200 mg/kg) of Sb resulted in a 5.63–22.78% reduction in aboveground biomass relative to that inoculated with inactive AMF, while the M+ group treated with Sb at the same concentration resulted in a 6.14–22.56% reduction in underground biomass relative to the M− group. In addition, significant linear correlations were observed between the concentration of Sb and biomass values of the M+ and M− groups (Figures 1C,D). The linear slope showed that, for both aboveground and underground biomass, the slope of the M+ group was significantly greater than that of the M− group. These results indicated that the heavy metal exposure levels in rice was the direct influencing factor leading to biomass reduction, and the addition of AMF could significantly aggravate this phenomenon. This is likely because AMF inoculation could make more Sb transfer from soil to the root of rice through mycelia, thus inhibiting the biomass of rice (Guo et al., 1996; Chen et al., 2003). This was observed in the microstructures of AMF infection on rice roots through the ink–vinegar staining method (Figure 2). In addition, our results found that inoculation with AMF inhibited plant growth in comparison with non-inoculated groups, the another possible reason may be that the cost of organic carbon obtained by AMF from plants was greater than that of other nutrients provided by AMF (Johnson et al., 1997; Liao et al., 2003; Citterio et al., 2005).

**FIGURE 3:**
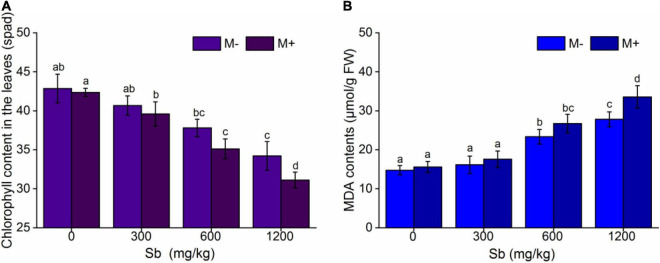
Effects of AMF on chlorophyll contents and MDA contents of rice leaf. **(A)** Chlorophyll content in the leaves, **(B)** MDA content in the leaves. FW represents fresh weight. Error bar was calculated from three parallel samples. Error bars sharing no common letter indicate that chlorophyll content and MDA content are significantly different at *P* < 0.05 level for treatments. The data are means ± SDs (*n* = 3).

AMF inoculation of rice significantly reduced chlorophyll content, and the chlorophyll content decreased with increasing concentrations of Sb (Figure 3 and Supplementary Table 3). Figure 3A showed that the chlorophyll content of the M+ groups was significantly lower than that of the M− groups at the same Sb concentration. Without heavy metal pollution, the chlorophyll content of the inoculated group was reduced by 1.17% compared with that of the non-inoculated group. When treated with a series dose (300, 600, and 1,200 mg/kg) of Sb, chlorophyll content was reduced 2.68–9.01% relative to those inoculated with inactive AMF rice (M− group). Chlorophyll is an important factor in plant photosynthesis, and its content directly affects the plant biomass (Felip and Catalan, 2000). Therefore, the effect of AMF inoculation on chlorophyll content in rice was consistent with that on biomass (Figure 1). This indicated that the growth of rice plants treated with Sb was further inhibited by AMF inoculation according to the decrease in chlorophyll content.

We further measured membrane lipid peroxidation (MDA), and the results were shown in Figure 3B and Supplementary Table 3. The MDA content in the M+ groups was higher than that in the M− groups (Figure 3B), which demonstrated that the inoculation of AMF accelerated the degree of membrane lipid peroxidation and increased chloroplast membrane breakage, resulting in chloroplast leakage. MDA is one of the final products of membrane lipid peroxidation caused by membrane structure breakage, which increases the content and further reflects the degree of membrane structure breakage, such as in the cell and chloroplast membranes (Sun et al., 2013; Wu et al., 2019).

### Effects of Arbuscular Mycorrhizal Fungi Inoculation on the Accumulation of Antimony in Rice

**FIGURE 4:**
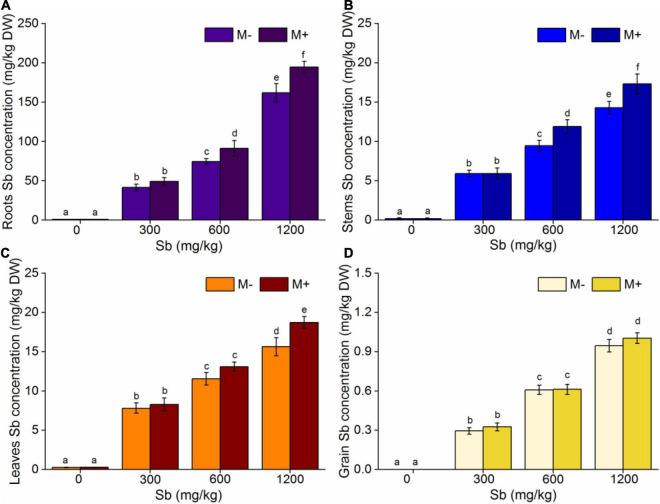
Effects of AMF on Sb concentrations of rice. **(A)** Roots Sb concentration, **(B)** stems Sb concentration, **(C)** leaves Sb concentration, **(D)** grain Sb concentration. Error bar was calculated from three parallel samples. Error bars sharing no common letter indicate that Sb concentration are significantly different at *P* < 0.05 level for treatments. The data are means ± SDs (*n* = 3).

The decrease of biomass and chlorophyll content of rice after AMF inoculation were mainly related to the change of Sb content and its existing form in rice after AMF inoculation. To verify the above hypothesis, we further studied the distribution and morphology of Sb in rice with or without AMF inoculation. The study found that Sb concentration of different tissues of rice increased with increasing Sb content, while inoculation with AMF accelerated the Sb absorption of rice at the same concentrations (Figure 4 and Supplementary Table 4). As shown in Figure 4, AMF had an insignificant effect on Sb absorption in rice at lower Sb concentrations (0 and 300 mg/kg). However, with the increase in Sb content, the concentrations of Sb in the AMF inoculated group was significantly higher than that in the non-inoculated group. When the concentration of Sb reached 600 mg/kg, the accumulation capacity of Sb in the roots, stems, and leaves of the M+ group was, respectively, 1.18, 1.21, and 1.13 times that of the M− group (Figures 4A–C). When the concentration of Sb increased to 1,200 mg/kg, the ability of AMF to enhance the metal accumulation for each rice part was further enhanced. The accumulation of Sb in the roots, stems, and leaves of the M+ group was, respectively, 1.20, 1.21, and 1.20 times higher than that of the M− group. Notably, the absorption ability of Sb for grain after inoculation with AMF was not obviously enhanced statistically (Figure 4D). This may be because Sb in the soil enters rice through the roots and migrates to different parts of the rice plant. The rice grains were the most link of the Sb migration and transfer pathway. Therefore, both the accumulation of Sb and the effect of AMF inoculation on the concentrations of Sb in grain were insignificant compared to those in other rice parts. Although AMF inoculation had no significant effect on the accumulation of Sb in grain, this negative effect still resulted in a significant decrease in the biomass of the grain and reduced the bioavailability of rice at high Sb concentrations (Supplementary Figure 2).

**FIGURE 5:**
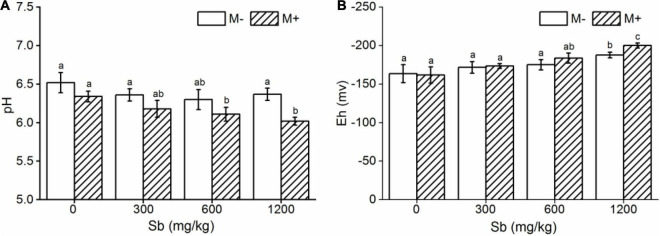
Effects of AMF on pH and Eh in rhizosphere soil. **(A)** pH, **(B)** Eh. Error bar was calculated from three parallel samples. Error bars sharing no common letter indicate that pH and Eh are significantly different at *P* < 0.05 level for treatments. The data are means ± SDs (*n* = 3).

**FIGURE 6:**
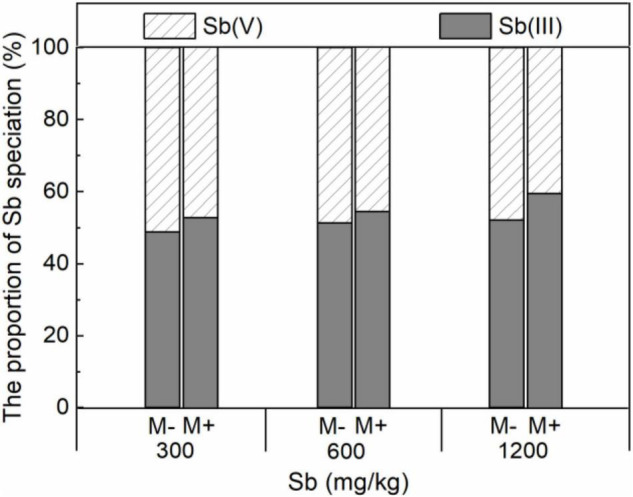
Effects of AMF on the proportion of Sb speciation in roots of rice.

Symbiotic nets can be formed between rice roots and extraradical mycelium of AMF, and the symbiotic net can extend the contact area of plants with Sb in the soil, making Sb available for uptake by rice roots (Cejpková et al., 2016). This phenomenon was also confirmed in our microscope experiment (Figure 2 and Supplementary Figure 3), which caused further metal entering the rice and accumulating in various plant parts under conditions of AMF inoculation. In addition, the change of physical and chemical properties of rhizosphere soil was another reason for accelerating Sb accumulation by AMF inoculation. First, the addition of AMF significantly increased the acidity of the soil (Figure 5A and Supplementary Table 5), which was due to the fact that AMF exude amino acids, acetic acid, and citric acid and activate the acid phosphatase of plants (Willamson and Alexander, 1975; Wei et al., 2016). The increase in soil acidity will promote the transformation of Sb from carbonate mineral to a soluble state, making it easier for Sb to enter various rice parts from the roots (He, 2007; Ning et al., 2015). Second, at the same time, inoculation with AMF can also significantly increase the electronegativity of the soil (Figure 5B), which directly affects the speciation state of the metalloid Sb in soil, in particular the conversion between Sb(V) and Sb(III), while the toxicity of Sb(III) was approximately 10-fold higher than the toxicity of Sb(V) (Chai et al., 2016). To further verify this phenomenon, we measured the content of different speciation of Sb in rhizosphere soil. As shown in Figure 6, when the amount of Sb added to the soil was 300, 600, and 1,200 mg/kg, the ratio of Sb(III) in the M+ group was 52.81, 54.54, and 59.52%, respectively. Compared with this, the ratio of Sb(III) in the M− group was 48.76, 51.28, and 52.12%, respectively, and lower than that in the M+ group. More Sb(III) was absorbed by rice than Sb(V) due to molecule configuration. Additionally, Sb(III) was in the form of neutral Sb(OH)_3_ in the soil, whereas Sb(V) was in the form of Sb(OH)_6_^–^ (Nakamaru and Altansuvd, 2014). Sb(OH)_3_ can enter the roots through aquaporins, which does not consume ATP (Feng et al., 2013). In contrast, plants might uptake Sb(OH)_6_^–^ through low selectively anion transporters, such as Cl^–^ and NO_3_^–^ transporters, which requires ATP (Tschan et al., 2009). Furthermore, the negatively charged cell wall would hinder the transportation of similarly charged Sb(OH)_6_^–^ to plants (Ren et al., 2014; Zhu et al., 2020). The combination of these factors made it easier for Sb(III) to enter rice than Sb(V). In conclusion, after inoculation with AMF, the contact area of rice root increased to absorb heavy metal, and soil properties changed. This influenced the presence of Sb, making it easier and more toxic to enter rice. These phenomena will lead to further enhancement of the metal poisoning in rice and cause a decrease in biomass, as shown in section of effects of AMF inoculation on biomass and chlorophyll content of rice.

### Effect of Arbuscular Mycorrhizal Fungi on Oxidative Stress Reactions in Rice

**FIGURE 7:**
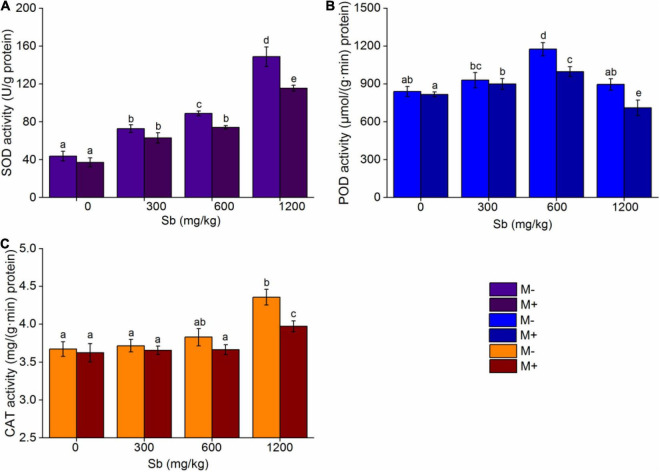
Effects of AMF on antioxidant enzymes of rice. **(A)** SOD, **(B)** POD, **(C)** CAT. Error bar was calculated from three parallel samples. Error bars sharing no common letter indicate that SOD, CAT, and POD activity are significantly different at *P* < 0.05 level for treatments. The data are means ± SDs (*n* = 3).

In order to further verify that AMF inoculation was mainly due to increasing the content of Sb in rice, thus increasing the stress effect of heavy metals on rice, we further examined the antioxidant enzyme activity of the M+ and M− groups. Further analysis of the antioxidant enzymes revealed that AMF inoculation increased the degree of heavy metal contamination in rice and led to weak growth (Figure 7). When the plant is in a stable state, the antioxidant enzymes in its body are in a dynamic balance. When the plant is attacked by harmful factors such as heavy metals, the antioxidant enzyme activity will increase to alleviate abiotic stress, so antioxidant enzyme activity is an important factor in assessing the response to heavy metal stress (Meghnous et al., 2019; Rajabpoor et al., 2019).

In the present study, we measured the changes of three typical antioxidant enzymes, including SOD, CAT, and POD, in rice with and without AMF inoculation. As shown in Figure 7 and Supplementary Table 3, with the increased concentration of Sb, the response activities of the three antioxidant enzymes increased to different degrees, while inoculation with AMF decreased the corresponding values of the activities of the three antioxidant enzymes compared to non-inoculation AMF group. This phenomenon indicated that inoculation with AMF inhibited rice from activating the antioxidant enzymes to respond to heavy metal stress. In addition, the response of different antioxidant enzymes was different. For SOD (Figure 7A), the enzyme activity increased linearly with the increase in Sb concentration, because SOD was the first enzyme to defend against reactive oxygen species (ROS), and it could reduce the conversion of superoxide radicals (${\text{O}}_{2}^{\cdot -}$) to hydrogen peroxide (H_2_O_2_) (Meier et al., 2011). When the concentration of Sb increased to 1,200 mg/kg, the enzyme activity of SOD was 3.40 times that of those without heavy metal addition, while the same enzyme activity of SOD was 3.11 times that without Sb addition after AMF inoculation. For POD (Figure 7B), the enzyme activity also increased linearly with the increase in Sb concentration; however, the increase in POD activity did not appear until the Sb concentration increased to 600 mg/kg, which may be because lower Sb concentrations could not activate the function of POD, which was to relieve the H_2_O_2_ produced by SOD (Bowler et al., 1992; Chai et al., 2016), so their activity response with the increase in metal concentration was behind that of SOD. Notably, the activity of POD for both the M+ and M− groups rapidly declined when the concentration of Sb was 1,200 mg/kg, because the tolerance of POD to Sb reached its limit, thus resulting in a significant decrease in enzyme activity (Hou et al., 2007; Chai et al., 2016). Interestingly, as shown in Figure 7C, when the concentration of Sb was below 1,200 mg/kg, the enzyme activity of CAT was not significantly changed, but when the concentration of Sb increased to 1,200 mg/kg, the enzyme activity of CAT increased rapidly. This may have occurred because the function of CAT and POD are to degrade hydrogen peroxide produced by SOD, and the response sensitivity of CAT may be poorer than that of POD (Kassa-Laouar et al., 2019). Therefore, when POD reached the upper limit of Sb tolerance, the stress response function of CAT was more activated. In addition, inoculation with AMF simultaneously reduced SOD, CAT, and POD activity in comparison to non-inoculated groups at the same Sb concentration, indicating that inoculation with AMF decreased antioxidant enzyme activity, resulting in more residual ROS in the leaves of rice, accelerating ROS damage to rice cells, and inhibiting rice growth.

## Conclusion

This research elucidated that AMF plays a negative role in Sb transport in soil–rice systems. The presence of AMF increased the uptake of Sb in rice, thus aggravating the invasion of heavy metals. As a result, the chloroplast membrane of plants rupture, resulting in reducing photosynthesis and eventually leading to a significant reduction in the biomass of all parts of the plant, aboveground and underground. This was further corroborated by the decreased activity of various antioxidant enzymes caused by the enhanced stress response. Consequently, the presence of AMF would accelerate the invasion of Sb in rice. These phenomena will help us better understand the migration and transformation of Sb in soil-rice system in real natural environment. Such altered contaminant-accumulating capacities may significantly affect the availability and food safety of rice. Our research will further focus on screening the related proteins of Sb transport and regulatory network of Sb membrane protein to elucidate the molecular mechanism of AMF affecting Sb uptake and transport in rice roots. This aspect should be given consideration in the assessment of the effect of AMF on rice uptake of Sb, avoiding the health risks of rice consumption.

## Data Availability Statement

The original contributions presented in the study are included in the article/Supplementary Material, further inquiries can be directed to the corresponding author/s.

## Author Contributions

MZ and XIL: conceptualization, methodology, data analysis and processing, visualization, and writing. XUL: sampling, investigation, and formal analysis. YM, RZ, HS, and HL: sampling and investigation. ZF: methodology and data analysis. YW and FW: research design, supervision, and funding acquisition. All authors contributed to the article and approved the submitted version.

## Conflict of Interest

The authors declare that the research was conducted in the absence of any commercial or financial relationships that could be construed as a potential conflict of interest.

## Publisher’s Note

All claims expressed in this article are solely those of the authors and do not necessarily represent those of their affiliated organizations, or those of the publisher, the editors and the reviewers. Any product that may be evaluated in this article, or claim that may be made by its manufacturer, is not guaranteed or endorsed by the publisher.
