# Supplementary material for: Effects of Antimony on Rice Growth and Its Existing Forms in Rice Under Arbuscular Mycorrhizal Fungi Environment
Source: Front Microbiol. 2022 Mar 22;13:814323. doi: 10.3389/fmicb.2022.814323 (PMC8981305; doi:10.3389/fmicb.2022.814323)
Supplement: Supplementary file 1 [file Data_Sheet_1.docx]

**Supplementary material**

**Effects of antimony on rice growth and its existing forms in rice under arbuscular mycorrhizal fungi environment**

Min Zhou ^1,2†^, Xinru Li ^2†^, Xuesong Liu ^2^, Yidong Mi ^1,2^, Zhiyou Fu ^2^,

Ruiqing Zhang ^3^, Hailei Su ^2^, Yuan Wei ^2,*^, Huifang Liu ^2^, Fanfan Wang ^2,*^

^1^ College of Environment, Hohai University, Nanjing 210098, China

^2^ State Key Laboratory of Environmental Criteria and Risk Assessment, Chinese Research Academy of Environmental Sciences, Beijing 100012, China.

^3^ School of Ecology and Environment, Inner Mongolla University, Hohhot 010040, China

^†^These authors have contributed equally to this work and share first authorship.

*** Correspondence:**

Corresponding author:

**Yuan Wei, Fanfan Wang**

E-mail address: [rbq-wy@163.com](mailto:rbq-wy@163.com) (Yuan Wei), [wwffaannaza@126.com](mailto:wwffaannaza@126.com) (Fanfan Wang).

TEL: +86-10-84931804.

**Supplementary Tables and Figures**

**Supplementary Tables**

**Table S1** Closed-vessel microwave digestion program.

**Table S2** The effect of AMF on biomass of different parts of rice.

**Table S3** The effect of AMF on antioxidant enzymes, MDA and Chlorophyll content of rice leaf.

**Table S4** The effect of AMF on Sb content of different parts of rice and Sb speciation of rice root.

**Table S5** The effect of AMF on pH, Eh of rhizosphere soil.

**Supplementary Figures**

**Fig S1** Pictorial view of rice after 100 days of growth in the pots.

**Fig S2** Effects of AMF on biomass of grain. Error bar was calculated from three parallel samples. Error bars sharing no common letter indicate that biomass of grain are significantly different at *P* < 0.05 level for treatments. The data are means ± standard deviations (SDs) (n = 3).

**Fig S3** Root colonization rate of rice by inoculated with AMF. Error bar was calculated from three parallel samples. Error bars sharing no common letter indicate that biomass of grain are significantly different at *P* < 0.05 level for treatments. The data are means ± standard deviations (SDs) (n = 3).

**Table S1** Closed-vessel microwave digestion program.

| Stage | Temperature (ºC) | Ramp (min) | Hold (min) |
| --- | --- | --- | --- |
| 1 | 120 | 8 | 10 |
| 2 | 160 | 5 | 20 |
| 3 | 195 | 5 | 90 |

**Table S2** The effect of AMF on biomass of different parts of rice.

| Group (mg/kg) | Inoculation or uninoculation | Biomass of Aboveground  (g/pot DW^*^) | Biomass of underground  (g/pot DW^*^) | Biomass of grain  (g/pot DW^*^) |
| --- | --- | --- | --- | --- |
| 0 | M- | 41.9 ± 2.8 a | 7.1 ± 0.8 a | 17.9 ± 0.6 a |
|  | M+ | 39.7 ± 4.1 ab | 7.0 ± 0.6 a | 17.6 ± 1.1 a |
| 300 | M- | 38.4 ± 3.7 ab | 6.4 ± 0.4 a | 17.2 ± 0.5 a |
|  | M+ | 36.2 ± 4.8 ab | 6.0 ± 0.5 ab | 16.8 ± 1.3 a |
| 600 | M- | 34.5 ± 1.9 b | 5.5 ± 0.3 b | 14.85 ± 0.8 ab |
|  | M+ | 30.1 ± 1.1 c | 4.7 ± 0.2 c | 14.1 ± 0.8 bc |
| 1200 | M- | 32.1 ± 2.9 bc | 4.6 ± 0.2 c | 14.2 ± 0.5 b |
|  | M+ | 24.8 ± 3.2 d | 3.6 ± 0.1 d | 12.6 ± 0.9 c |

* DW represents dry weight. There is no common letter indicate that biomass of different parts of rice are significantly different at *P* < 0.05 level for treatments.

**Table S3** The effect of AMF on antioxidant enzymes, MDA and Chlorophyll content of rice leaf.

| Group (mg/kg) | Inoculation or uninoculation | SOD  (U/g protein) | CAT  μmol/(g·min) | POD  mg/(g·min) | MDA  μmol/mg FW^*^ | Chlorophyll content |
| --- | --- | --- | --- | --- | --- | --- |
| 0 | M- | 43.77 ± 5.09 a | 3.67 ± 0.10 a | 840.89 ± 39.00 ab | 14.75 ± 1.17 a | 42.85 ± 1.84 ab |
|  | M+ | 37.13 ± 4.65 a | 3.62 ± 0.12 a | 817.01 ± 19.30 a | 15.58 ± 1.43 a | 42.35 ± 0.52 a |
| 300 | M- | 72.70 ± 4.05 b | 3.72 ± 0.08 a | 929.84 ± 60.73 bc | 16.14 ± 2.22 a | 40.67 ± 1.24 ab |
|  | M+ | 62.90 ± 5.47 b | 3.66 ± 0.06 a | 899.73 ± 42.00 b | 17.56 ± 2.14 a | 39.58 ± 1.56 b |
| 600 | M- | 88.85 ± 2.52 c | 3.83 ± 0.11 a | 1175.25 ± 51.72 d | 23.36 ± 1.86 b | 37.80 ± 1.12 bc |
|  | M+ | 74.09 ± 1.89 b | 3.66 ± 0.07 a | 996.95 ± 38.61 c | 26.72 ± 2.37 bc | 35.12 ± 1.29 c |
| 1200 | M- | 148.73 ± 10.29 d | 4.36 ± 0.10 b | 896.03 ± 45.10 ab | 27.80 ± 1.92 c | 34.20 ± 1.84 c |
|  | M+ | 115.56 ± 3.11 e | 3.97 ± 0.07 c | 710.77 ± 60.87 e | 33.57 ± 2.89 d | 31.12 ± 1.02 d |

* FW represents fresh weight. There is no common letter indicate that SOD, CAT, POD, MDA and Chlorophyll content are significantly different at *P* < 0.05 level for treatments.

**Table S4** The effect of AMF on Sb content of different parts of rice and Sb speciation of rice root.

| Group (mg/kg) | Inoculation or uninoculation | Root Sb concentration (mg/kg) | Stem Sb concentration  (mg/kg) | Leaf Sb concentration  (mg/kg) | grain Sb  concentration  (mg/kg) | Sb^3+^ of rice root | Sb^5+^ of rice root |
| --- | --- | --- | --- | --- | --- | --- | --- |
| 0 | M- | 0.79 ± 0.10 a | 0.19 ± 0.10 a | 0.26 ± 0.03 a | 7.63×10^-4^ ± 1.52×10^-5^ a | None | None |
|  | M+ | 0.80 ± 0.05 a | 0.19 ± 0.09 a | 0.28 ± 0.02 a | 9.82×10^-4^ ± 1.47×10^-5^ a | None | None |
| 300 | M- | 41.42 ± 4.23 b | 5.92 ± 0.41 b | 7.83 ± 0.67 b | 0.29 ± 0.02 b | 18.73 ± 3.71 a | 19.69 ± 4.98 a |
|  | M+ | 49.17 ± 4.72 b | 5.92 ± 0.71 b | 8.31 ± 0.83 b | 0.33 ± 0.03 b | 23.33 ± 4.25 a | 20.84 ± 3.34 a |
| 600 | M- | 74.50 ± 3.55 c | 9.47 ± 0.68 c | 11.55 ± 0.80 c | 0.61 ± 0.03 c | 36.67 ± 3.87 b | 34.83 ± 5.01 b |
|  | M+ | 91.30 ± 9.80 d | 11.9 ± 0.86 d | 13.11 ± 0.58 c | 0.61 ± 0.04 c | 49.80 ± 6.02 b | 41.50 ± 4.74 b |
| 1200 | M- | 161.85 ± 11.65 e | 14.28 ± 0.81 e | 15.64 ± 1.15 d | 0.95 ± 0.05 d | 74.81 ± 8.99 c | 87.04 ± 6.16 c |
|  | M+ | 194.57 ± 7.47 f | 17.33 ± 1.26 f | 18.73 ± 0.75 e | 1.00 ± 0.04 d | 115.81 ± 12.17 d | 78.76 ± 3.72 c |

There is no common letter indicate that Sb concentration and Sb valence are significantly different at *P* < 0.05 level for treatments. “None” represents undetected.

**Table S5** The effect of AMF on pH, Eh of rhizosphere soil.

| Group (mg/kg) | Inoculation or uninoculation | pH | Eh (mv) |
| --- | --- | --- | --- |
| 0 | M- | 6.53 ± 0.13 a | -163.7 ± 11.6 a |
|  | M+ | 6.34 ± 0.05 a | -161.9 ± 10.4 a |
| 300 | M- | 6.36 ± 0.08 a | -171.8 ± 7.5 a |
|  | M+ | 6.18 ± 0.25 ab | -173.6 ± 3.2 a |
| 600 | M- | 6.30 ± 0.16 ab | -175.2 ± 6.7 a |
|  | M+ | 6.11 ± 0.09 b | -183.8 ± 6.6 ab |
| 1200 | M- | 6.37 ± 0.08 a | -187.8 ± 3.7 b |
|  | M+ | 6.02 ± 0.05 b | -200.1 ± 3.2 c |

There is no common letter indicate that pH and Eh are significantly different at *P* < 0.05 level for treatments.


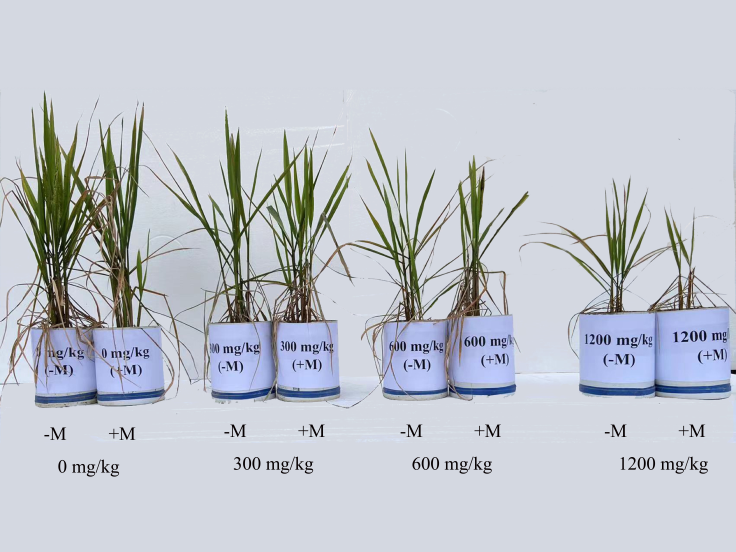


**Fig S1** Pictorial view of rice after 100 days of growth in the pots.





**Fig S2** Effects of AMF on biomass of grain. DW represents dry weight. Error bar was calculated from three parallel samples. Error bars sharing no common letter indicate that biomass of grain are significantly different at *P* < 0.05 level for treatments. The data are means ± standard deviations (SDs) (n = 3).





**Fig S3** Root colonization rate of rice by inoculated with AMF. Error bar was calculated from three parallel samples. Error bars sharing no common letter indicate that biomass of grain are significantly different at *P* < 0.05 level for treatments. The data are means ± standard deviations (SDs) (n = 3).
