# Supplementary figures and images for: Effects of Antimony on Rice Growth and Its Existing Forms in Rice Under Arbuscular Mycorrhizal Fungi Environment
Source: Front Microbiol. 2022 Mar 22;13:814323. doi: 10.3389/fmicb.2022.814323 (PMC8981305; doi:10.3389/fmicb.2022.814323)

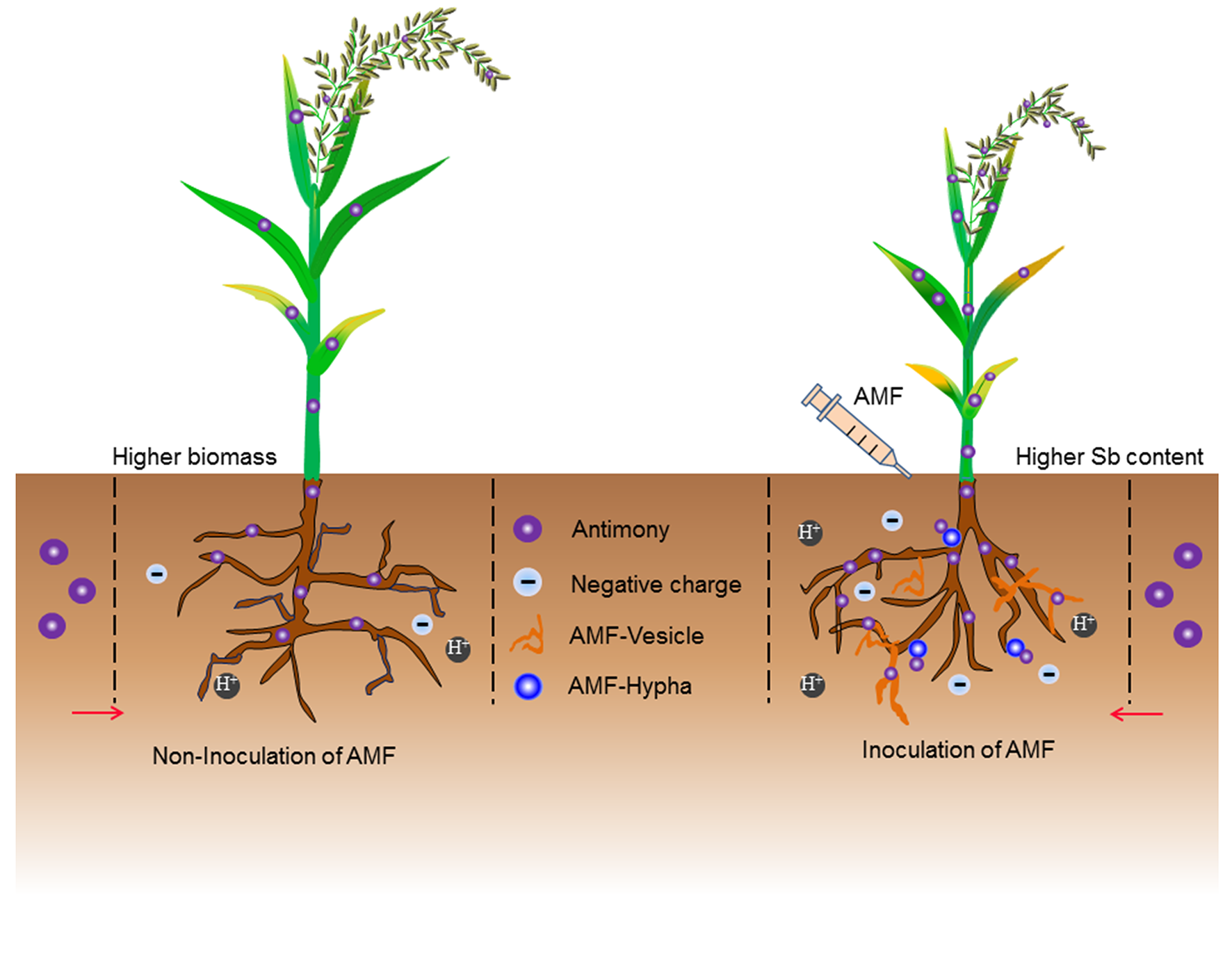

Supplement: Supplementary file 2 [file Image_1.TIF]
